# Exploring ecological modelling to investigate factors governing the colonization success in nosocomial environment of *Candida albicans* and other pathogenic yeasts

**DOI:** 10.1038/srep26860

**Published:** 2016-06-01

**Authors:** Laura Corte, Luca Roscini, Claudia Colabella, Carlo Tascini, Alessandro Leonildi, Emanuela Sozio, Francesco Menichetti, Maria Merelli, Claudio Scarparo, Wieland Meyer, Gianluigi Cardinali, Matteo Bassetti

**Affiliations:** 1 Department of Pharmaceutical Sciences-Microbiology, University of Perugia, Borgo 20 Giugno 74, 06121 Perugia, Italy; 2 U.O. Malattie Infettive, Azienda Ospedaliera Universitaria Pisana, Via Paradisa 2, Cisanello, 56100 Pisa, Italy; 3 U.O. Medicina d’Urgenza (Emergency Medicine Unit) Universitaria, Azienda Ospedaliera Universitaria Pisana, Via Paradisa 2, Cisanello, 56100 Pisa, Italy; 4 Infectious Diseases Clinic, Santa Maria Misericordia University Hospital, Piazzale Santa Maria della Misericordia, 15, 33100 Udine, Italy; 5 Microbiology Unit, Santa Maria Misericordia University Hospital, Piazzale Santa Maria della Misericordia, 15, 33100 Udine, Italy; 6 Molecular Mycology Research Laboratory, Centre for Infectious Diseases and Microbiology, Sydney Medical School – Westmead Hospital, Marie Bashir Institute for Infectious Diseases and Biosecurity, The University of Sydney, Westmead Institute for Medical Research, Sydney, Australia; 7 CEMIN, Centre of Excellence on Nanostructured Innovative Materials, Department of Chemistry, Biology and Biotechnology, University of Perugia, Via Elce di Sotto 8, 06123 Perugia, Italy

## Abstract

Two hundred seventy seven strains from eleven opportunistic species of the genus *Candida*, isolated from two Italian hospitals, were identified and analyzed for their ability to form biofilm in laboratory conditions. The majority of *Candida albicans* strains formed biofilm while among the NCAC species there were different level of biofilm forming ability, in accordance with the current literature. The relation between the variables considered, i.e. the departments and the hospitals or the species and their ability to form biofilm, was tested with the assessment of the probability associated to each combination. Species and biofilm forming ability appeared to be distributed almost randomly, although some combinations suggest a potential preference of some species or of biofilm forming strains for specific wards. On the contrary, the relation between biofilm formation and species isolation frequency was highly significant (R^2^ around 0.98). Interestingly, the regression analyses carried out on the data of the two hospitals separately were rather different and the analysis on the data merged together gave a much lower correlation. These findings suggest that, harsh environments shape the composition of microbial species significantly and that each environment should be considered *per se* to avoid less significant statistical treatments.

*Candida* bloodstream infection (BI) is an important cause of morbility and mortality in health care settings and represents the fourth cause of nosocomial sepsis in the USA and in most developed countries^1^,^2^. Candidemia is responsible for unacceptable percentages of attributable and overall mortality rate ranging from 30–81% and from 5–71%^3^, respectively. The incidence of candidemia rose in the last decades of the 20^th^ century due to several risk factors^4^. *Candida albicans* remains the most common species causing BI, followed by several “non *Candida albicans Candida* species” (NCAC) among which *Candida glabrata, Candida parapsilosis* and *Candida tropicalis* are usually the most common and show increasing incidence^5^,^6^,^7^. The various species are a further factor to interpret the origin of infection, in fact *C. albicans, C. glabrata* and *C. tropicalis* are considered predominantly commensal and therefore more present in cases of endogenous infections. On the contrary, species found in the natural and anthropic environment, as *C. parapsilosis*, *Meyerozyma guilliermondii* (telomorph of *C. guilliermondii*) and *Wickerhamomyces anomalus*, are more present in exogenous infections^8^. The recent finding that medical and food isolates of *M. guilliermondii* cluster differently, according to the ITS and LSU markers, poses the question on whether the exogenous infection is caused by strains of the nosocomial environment or of other niches^9^. The increasing frequency of NCAC species has been extensively reported in the last years^4^,^6^,^10^,^11^,^12^,^13^ with significant epidemiological and ecological differences among various geographic areas^7^,^14^. This situation represents a serious threat, complicated by a significantly lesser knowledge of biofilm and resistance mechanisms in NCAC then in *C. albicans.*

The ability of some fungal species to form biofilm is considered an important factor for their persistence on medical devices^15^,^16^, and in general in the nosocomial environment^17^, however, the actual extent of the fungal persistence in the hospital is still unclear. Furthermore, the fungal biofilm displays higher resistance to drugs^18^,^19^,^20^, with a complex and yet not totally understood mechanism^7^, involving the Hsp90, a protein also responsible for cell dispersion^21^, making the biofilm a system to persist in harsh environments and resist to the associated stresses.

From what briefly reported above, the biofilm forming ability represents a severe risk factor^1^,^22^ and adds more problems to the need of a timely and appropriate therapy to reduce the mortality^23^.

Altogether, *Candida* infections represent a serious problem and their ability to form biofilm seems to represent not only a medical, but also an ecological problem. In fact, the infecting cells can be present in different niches spanning from the devices to the surfaces, the air, some foods and the patients themselves. The cell circulation in the environment is an essential point to understand the complex ecology represented by the interaction of fungal cells with patients, different substrates and drugs. Furthermore, only a good ecological insight can lead to the actual possibility of “catching” these pathogens in their actual niches before the infection. In fact, once the infection occurred there are relatively few therapeutics to treat these diseases successfully, whereas environmental treatments with harsh biocidal compounds can be effective and decrease significantly the incidence and the mortality caused by these fungi.

With the above rationale, the present study has been designed around two hypotheses: *i.* the hospital and the various departments, i.e. specific environments, are key factors for the frequency of candidemias; *ii.* the ability to form biofilm has a measurable effect on the incidence of these diseases.

For this purpose, 277 strains of eleven *Candida* species have been isolated from the various departments of two Italian hospitals (Pisa and Udine) 450 km apart, identified at the species level and tested for biofilm formation.

## Results

### Distribution of the studied characters

#### Species in the hospitals and wards

**Figure 1:**
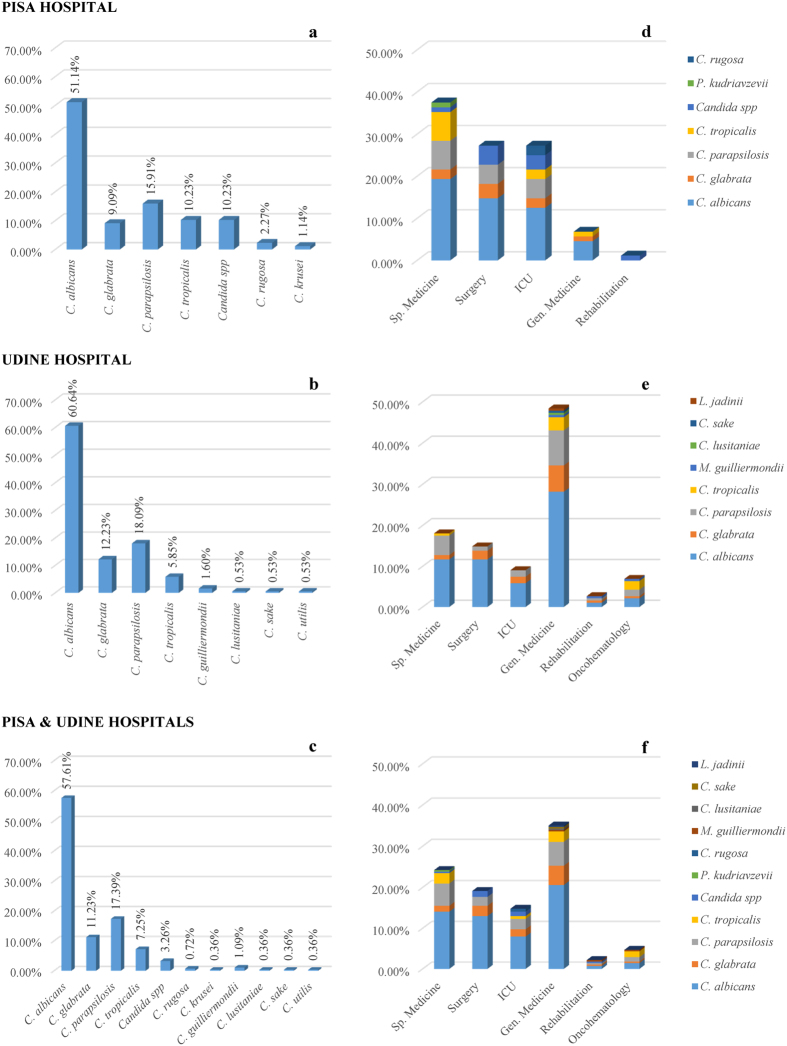
Species presence in the two hospitals studied and relatively frequency of isolation of the different species in the hospitals wards.

Four species were isolated in both hospitals: *C. albicans, C. glabrata, C. parapsilosis* and *C. tropicalis* (Fig. 1, panel a,b). In the Pisa hospital were isolated specifically *Pichia kudriavzevii* (telomorph of *Candida krusii*)*, C. rugosa* and some strains yet to be attributed to a possibly new yeast species (hereinafter referred to as *Candida. spp*) (panel a). The species isolated uniquely in Udine were *Clavispora lusitaniae* (telomorph of *Candida lusitaniae*)*, C. sake, Cyberlindnera jadinii* (telomorph of *Candida utilis*) and *Meyerozyma guilliermondii* (telomorph of *Candida guilliermondii*) (panel b).The four most represented species present in both hospitals accounted for 86.36% and 96.81% in Pisa and Udine hospitals, respectively. As expected, *C. albicans* was the most frequently isolated species (panel c), absent only from the Rehabilitation department of Pisa (panel d). The second most frequent species was *C. parapsilosis,* followed by *C. glabrata* and *C. tropicalis*. These data are in good agreement with the current literature^24^,^25^. The departments with the highest incidences were the specialized medicine, Surgery and ICU in Pisa, while general medicine in Udine was by far the ward with more isolations, followed by specialized medicine, Surgery and ICU (panel d,e). Merging those data produced a picture very similar to that described for Udine from which came 188 out of the 276 strains analyzed in this study (panel f).

#### Biofilm forming strains in hospital departments

**Figure 2:**
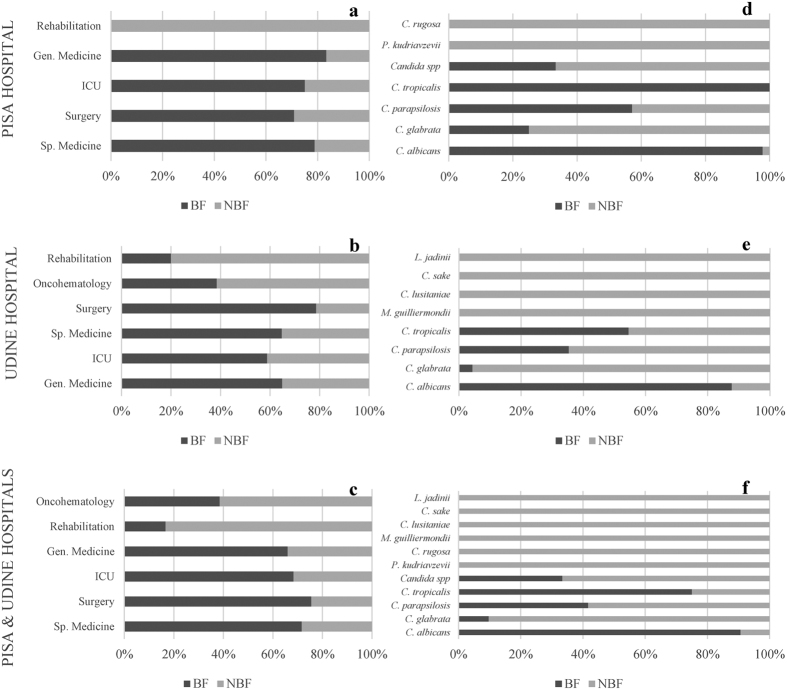
Relative occurrence of biofilm forming capability in different hospital departments and distribution of biofilm forming strains among species.

The various departments in the two hospital under study showed different frequencies of biofilm forming strains (Fig. 2). Namely, in Pisa the Rehabilitation ward had only non-biofilm forming isolates, whereas the other four departments showed frequencies ranging from 60 to more than 80% (panel a). The situation in Udine was more variable with frequencies of biofilm forming strains ranging from 20% (Rehabilitation) to 80% (Surgery) (panel b). Merging the data from the two hospitals gave a synthetic view with biofilm forming strain frequency ranging from less than 20% (Rehabilitation) to the 75% of the Surgery departments (panel c).

#### Biofilm forming strains and species

The four prominent species (*C. albicans, C. glabrata, C. parapsilosis* and*C. tropicalis*) showed different levels of biofilm forming ability, whereas the lesser present species (*C. rugosa*, *P. kudriavzevii*, *C. jadinii, C. sake, C. lusitaniae* and *M. guilliermondii*) did not produce biofilm (Fig. 2, panel f). *C. albicans* biofilm forming strains (hereinafter referred to as BF, in contrast with non BF referred to as NBF) were 97.78% and 87.71% in Pisa and Udine, respectively (panels d,e). Since these figures are particularly high, the biofilm formation test was repeated with two different methods using XTT and Tetrazonium Blu as indicators, obtaining no significant differences (data not shown). *C. glabrata* showed quite different frequencies of BF strains in Pisa (25%) and in Udine (4.3%), similarly *C. tropicalis* had all BF strains in Pisa and some 55% in Udine. Finally, *C. parapsilosis* showed 35% and 57% in Udine and Pisa, respectively. In general, Pisa had 75% BF strains and Udine 63.63%.

### Contingency analysis studied characters

#### Species vs. hospital departments

Different opportunistic yeast species can be distributed randomly in the various hospital departments or show specific preferences for the environment represented by the various wards. In order to test the null hypothesis, i.e. that species are distributed randomly within the hospital, a two-way contingency analysis was carried out, following the rationale described in Legendre & Legendre^26^, as outlined in the Experimental Procedures section. This analysis is the gold standard in ecological studies when non continuous qualitative data are employed, as in our case, and allows to calculate the probability associated to the *χ*^*2*^ (*p-*value) to accept or reject that the association between species and hospital department are independent. When two descriptors are non-independent, then their combination is indicative of some sort of specific occurrence. This test was carried out considering the two hospitals separately and then by merging all data for a joint analysis (Table 1).

The general *χ*^*2*^ test for the two-way contingency table, obtained with the data of the Pisa hospital, showed no statistical significance (*p* = 0.32), indicating that the distribution of the yeast species was largely due to random effect. However, the frequency of *C. rugosa* in the ICU, and of *Candida. spp* in the Rehabilitation ward, were significantly non independent with 0.0489 and 0.0049 *p*-values, respectively. These data suggested that these species have a certain level of specificity for these environments. Almost significant (*p* = 0.11741) was the frequency of isolation of *C. tropicalis* in the Surgery department (panel a).

The general *χ*^*2*^ test carried out with all the data from the Udine hospital gave 0.1189 *p*-value indicating independence between species and wards, assuming *p* = 0.10 as the minimum for statistical significance. However, the frequency of isolation of *M. guilliermondii* in Oncohematology and Rehabilitation showed high *χ*^*2*^ values corresponding to *p* = 0.081 and *p* = 0.0011, respectively.

*C. tropicalis* in Onchohaematology showed a significantly non-independence with *p* = 0.0002. Interestingly, three species (*C. albicans, C. parapsilosis, C. tropicalis*) in the Surgery ward displayed elevated *χ*^*2*^ values with *p* values ranging from 0.17 to 0.22, indicating that their frequency of isolation cannot be entirely ascribed to a randomness (panel b).

Merging the data of the two hospitals, the general *χ*^*2*^ test was significant with *p* = 0.00819, indicating that the hypotheses of random distribution of the species in the departments of the two hospitals can be rejected. In fact, several species-department combinations showed high and significant *χ*^*2*^ values, such as *C. tropicalis* in Surgery and Oncohaematology and *M. guilliermondii* in Rehabilitation and Oncohaematology (panel c). The difference between the *χ*^*2*^ tests of the two hospitals considered separately and then jointly can be ascribed to the fact that merging all data together produces larger numbers and a consequently more solid statistics. Interestingly, expectable random effects explain the frequency of isolation of the various species (null hypothesis) only in the specialized medicine departments. *C. tropicalis* was overrepresented in Oncohaematology and underrepresented in the Surgery department (*p* = 0.0504 and 0.0016 respectively). *M. guilliermondii* was isolated with a frequency higher than expected in Rehabilitation and Oncohaematology (*p* = 0.0002 and 0.022 respectively), although the absolute frequencies are low, that is typical for this rather ubiquitous species found on fruit^9^.

Most of the cases in which the frequency of isolation were different than expected (null hypothesis rejected) interested mostly the less representative species which account for ca. 13% in Pisa and 3% in Udine Hospitals. Based on this observation, the cases of statistically supported over- and under-isolation represent only few cases, maybe of little epidemiological importance. Yet these preferences are worth better insight, because these few cases could underline yet to be described phenomena of preferential environmental (nosocomial) colonization.

#### Biofilm forming strains vs. hospital departments

The question on whether the biofilm forming strains can dwell preferentially in some hospital departments was addressed with the same contingency approach as above (Table 1). The general *χ*^*2*^ test for the two-way contingency table, obtained with the data of the Pisa hospital, showed no statistical significance (*p* = 0.44), once again indicating that the combination of biofilm forming strains and hospital departments were largely due the randomness. In fact, no combination showed *p* values below 0.10 (panel d). On the contrary, in the Udine hospital, the *χ*^*2*^ test was associated with *p* = 0.0607, indicating that the frequencies of biofilm forming strains and the various departments of isolation cannot be considered totally independent. Although no combination showed significant *p* values below 0.10, some weak signal of non-independence existed for those departments with less biofilm to isolates as in Rehabilitation and Oncohaematology of the Udine hospital (p = 0.11 and 0.14, respectively) (panel e).

Merging the two datasets led to a two-way contingency table for which the hypothesis of independence cannot be rejected (*p* = 0.14). In general, these tests indicated the frequency of isolation of biofilm forming strains is based on the randomness, with some significant non independence for the combinations of the Udine hospital presented above (panel f).

#### Biofilm forming strains vs. species

The *χ*^*2*^ analysis of two-way contingency tables between the frequency of biofilm forming strains and species allowed to reject the null hypothesis with *p* = 3 × 10^−8^, 8.7 × 10^−6^ and 7.2 × 10^−22^ for Pisa, Udine and merged data, respectively. This extremely high statistical significance indicates that the frequency of isolation of the single species is influenced by their ability to form biofilm. More specifically, in the Pisa hospital, the random effect could be excluded for the BF strains of *Candida spp.* and for the NBF isolates of *C. glabrata, C. tropicalis* and *Candida. spp*. Particularly interesting the fact that *C. glabrata* had three times more NBF than expected on the basis of a random effect. In Udine, BF and NBF strain frequencies were significantly associated with *C. albicans, C. parapsilosis* and *C. glabrata*. Namely, *C. albicans* had 28% more BF than expected, while *C. glabrata and C. parapsilosis* had respectively 14 times more and 50% less BF strains than expected. The situation of both hospitals considered together was substantially similar to that of Udine, given the preponderance of the strains deriving from that hospital.

### Modelling the biofilm vs. isolation frequencies

The biofilm formation frequency was compared with the frequency of isolation of the single species because the combination of these two characters were shown to be non-random with extremely high statistical significance. These two descriptors had 98.51% and 97.37% Pearson correlation (R) in Pisa and Udine hospitals, respectively. This was confirmed by the linear correlation analysis with R^2^ of 97.04%, and 94.8% for Pisa and Udine, but with two totally different correlation equations. In fact, in Udine, the correlation curve could be described by the equation


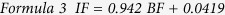


in which IF and BF indicate, respectively, the isolation frequency of single species and the biofilm formation frequency. Formula 3 specifies that the frequency of isolation of each species is 94.2% of the frequency of BF strains of the same species.

In Udine, the correlation analysis produced the same R^2^ as in Pisa, but a different equation:


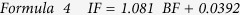


This equation indicates that in the Udine hospital the frequency of isolation of each yeast species is 108% of its frequency of BF strains. This observation indicates that indeed the ability to form biofilm plays a key role in the presence of the species in the hospital environment, but with different dynamics in Pisa and Udine. It was therefore not surprising that the correlation analysis of the Pisa and Udine data merged together produced an intermediate situation, described by Formula 5 with a lower R^2^ (60.94%) than those obtained with the two hospitals analyzed separately.


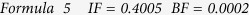


The differences between the two hospitals can be pin-pointed by comparing the IF/BF ratio of the single species. *C. albicans* showed 103% and 114% IF/BF ratios in Pisa and Udine respectively. This means that, given a frequency of BF *C. albicans* strains, the probability of isolating this species in Pisa is slightly lower than in Udine. *C. parapsilosis* had 175% and 283% IF/BF ratios in Pisa and Udine, showing that the biofilm forming ability influences the frequency of isolation more in Udine than in Pisa. A similar situation was observed for *C. tropicalis* with 100% and 183% IF/BF ratios in Pisa and Udine. Finally, *C. glabrata* showed 400% and 2300% IF/BF ratios in the two hospitals, making it the species for which the biofilm formation ability has the maximum effect on the frequency of isolation.

## Discussion

The distribution of opportunistic species of the genus *Candida* presented in this work is largely in accordance with the current literature. In fact, the number of isolates of *C. albicans* ranged from 51–61% in Pisa and Udine, showing figures similar to those previously reported^5^,^25^. *C. tropicalis* and *C. parapsilosis* showed 12.5% average frequency and *C glabrata* 10.6% with a significant difference from the 20% of the formers and the 5% of the latter recently reported in Brazil^7^. The seven species isolated in only one of the two hospitals showed low frequencies around 1%, again in accordance with most of the current literature^27^. These differences can be justified by the different geographic places where the studies were carried out, and by a general increase of NCAC species vs *C. albicans*^14^, indicating that epidemiological data differ significantly over the time and the geography. The question on how the geography and the hospital ward influence these species frequencies is difficult to address, due to a literature concentrating either on aggregated data or on specific wards, with very few papers reporting the distribution within the hospital. This suggests that more detailed reports will be necessary in future to track these aspects.

*Candida* infections have an endogenous origin based on the growth of the cells already dwelling on or in the body of the patient as a commensal, while the exogenous origin derives from the surrounding environment^28^. Early studies on these aspects suggested that different areas of the hospital can be more interested to one of the two types of *Candida* infection^29^. Exogenous nosocomial infections can be triggered by the ability of these fungi to persist in the environment, by forming biofilm. Kramer and colleagues^30^ reported ca 4 months persistance for *C. albicans* and *C. glabrata*, whereas these figures dropped to a few days according to an older study^31^. The persistence of *C. parapsilosis* was estimated on a couple of weeks by the above two studies. There is currently little if any information on whether the cells move as pieces of biofilm (sessile cells) or as planktonic cells liberated during the maturation of the biofilm and in their way to colonize another surface, initiating a new biofilm plaque^32^. In both cases, dispersed cells have showed enhanced adherence and produce a more robust biofilm than planktonic cells not deriving from a biofilm^33^. The biofilm architecture and resistance varies among species^34^,^35^,^36^,^37^ and sometimes with the genetic setting^38^, making any generalization quite difficult. The whole situations seems to be further complicated by the diversity of resistance mechanisms in young and mature biofilms, whereas the former is relatively well elucidated and based on multidrug pumps, the latter seems based on a dormancy mechanism leading to the production of “persister” cells^5^,^24^,^25^,^39^. The fungal biofilm displays higher resistance to drugs^18^,^19^,^20^, with a complex and yet not totally understood mechanism^7^, involving the Hsp90, a protein also responsible for cell dispersion^21^. The double function of this protein suggests that the complex regulation of the biofilm formation^40^ is responsible for dissemination and resistance: two key factors for the success of these cells in the hospital environment.

The Biofilm forming ability has been suggested as one of the major risk factors for mortality due to *C. albicans*^1^ and in general to the other yeast species analyzed in this article^7^. However, it seems that, in some settings, the biofilm forming ability does not increase the mortality nor the probability of getting catheter related candidemia^16^.

The study of the factors facilitating the formation of biofilm is an important and little investigated aspect^22^ to understand whether the biofilm formation is triggered by the environmental conditions. In this paper, we have considered different hospital departments to test if they have an influence in the biofilm formation. The contingency analysis has shown that there is no differential effect of the various departments in the presence of biofilm forming strains. Some instances, such as the Oncohematology of Udine, showed less biofilm than expectable. The statistical significance of these variations is relatively good when considering the aggregated data of the two hospitals (*p* = 0.031), whereas there is not significance when considering the disaggregated data of Udine alone. This suggests that we have only hints of an effect exerted by some departments, but higher number of strains for each hospital will be necessary for a careful determination of the statistical significance.

In our analytical conditions, *C. albicans, C. tropicalis, C. parapsilosis* and *C. glabrata* formed biofilm in 90%, 75%, 42% and 10% of the isolates with 67% among all the studied strains. These figures are in agreement with a recent study carried out in Scotland, in which the biofilm formation was reported as quartiles of the spectrophotometric quantification after Crystal Violet staining^1^. The high and intermediate producers accounted for 67%, 59 and 100% of the *C. albicans, C. tropicalis* and *C. parapsilosis* strains, whereas in both cases *C. glabrata* produced biofilm poorly. Interestingly, the high and intermediate producers reported for these three species in this study match exactly with the 67 of biofilm formers of our investigation. The differences in the portion of biofilm formers within the various species can be due to different environmental conditions.

These high levels of BF vs NBF strains can be due to at least three causes: the environment or the niche, methods and clonality. The study of the environmental effect is of great importance in these infections, very often caused by exogenous environmental contaminations. The other two factors, briefly outlined below, can seriously interfere with the effective determination of environmental effects on biofilm formation. The Crystal Violet, the XTT and the SYTO9 methods showed correlations ranging from 0.8 to 0.4, meaning poor R^2^ regression values ranging from 0.64 to 0.16^1^. This fact indicates that the three methods yield rather independent measures of biofilm formation and the results obtained with different methods can diverge significantly. The clonality can account for the same strain being isolated repeatedly as different isolates. This problem has not an easy solution, because very accurate analyses are necessary to rule out the hypothesis of two strains being identical. Even the current barcode marker ITS^41^ is scarcely effective differentiating isolates and combinations with more marker genes can be necessary to address the clonality problem effectively^9^,^42^. Until more light will not be shaded on this issue, we can only state that the isolates of these studies are very frequently able to form biofilm, regardless of their genetic relationships.

In the two hospital studied, the ability to form biofilm was directly correlated with the different frequency of the various isolated species according to solid linear correlations. The regression between species frequencies and biofilm formation studied separately in the two hospitals had better R^2^ (0.97 and 0.95 for Pisa and Udine, respectively) than when all data were merged together (R^2^ = 0.6).

This evidence indicates that the dynamics governing yeasts biofilm presence in the two hospitals considered are quite different. Although this situation cannot be generalized on the basis of a single study, care should be taken in epidemiological studies to analyze the data pertinent to the specific place from which they derive by condensing data because merging data from different environments (hospitals, cities etc.) might be induce serious bias.

Biofilm formation is considered of paramount importance in medicine and in a number of environmental applications. Altogether, this study has demonstrated that, in the two hospitals analyzed, the biofilm is the major factor triggering the persistence of the yeast species in these environments. A number of questions are still open, as the problem of clonality and the definition of a comprehensive working model to explain the role played by biofilm in persistence, resistance and spreading of the cells in the four most common opportunistic pathogens of the genus *Candida.*

The finding that biofilm formation can be an important factor to favor the presence of microbial cells in harsh environments further improves its general and applied biological importance.

## Methods

### Strains and growth condition

277 strains belonging to opportunistic species of the *Candida* genus, isolated in a clinical (medical) environment, were employed in this study (Tables S1 and S2, Supplementary materials). All strains were isolated from patient bloodcultures, with the exception of *C tropicalis* 6184a and 6184b isolated from peritoneal fluid, *C. parapsilosis* 6551 from pharyngeal swab and *C. albicans* 8158 and 8158/C from vascular prosthesis. Isolates are kept frozen at −80 °C in 17% glycerol. Short term storage was carried out on YEPDA (YEPD added with 1.7% agarose) at 4 °C. Strains were grown in YEPD (Yeast Extract 1%, Peptone 1%, dextrose 1% - all products from Biolife- http://www.biolifeitaliana.it/) at 37 °C with 150 rpm shaking.

### Molecular analysis ITS and bioinformatics tools

Genomic DNA was extracted as indicated by Cardinali *et al.*^43^. ITS1, 5.8S, ITS2 rDNA genes were amplified with FIREPol^®^ Taq DNA Polymerase (Solis BioDyne, Estonia), using ITS1 (5′-TCCGTAGGTGAACCTGCGG) - ITS4 (TCCTCCGCTTATTGATATGC) primers.

The amplification protocol was carried out as follows. Initial denaturation at 95 °C for 4 min, 35 amplification cycles (94 °C for 1 min, 53 °C for 1 min and 72 °C for 1 min) and final extension at 72 °C for 10 min. Amplicons were purified with the GFX PCR DNA purification kit (GE Healthcare) and subject to electrophoresis on 1.5% agarose gel (Gellyphor, EuroClone, Italy). Amplicons were sequenced in both directions with ABI PRISM technology by MACROGEN (www.macrogen.com) with the same primers used for the generation of the amplicons. Consensus sequences for each strain and trimming of the ends with low sequencing quality were carried out with Geneious R6 (v. 6.17, Biomatters, Auckland, New Zealand, www.geneious.com). ITS-based species identification was carried out with BLAST search^44^ in GenBank (www.ncbi.nlm.nih.gov/genbank/) and refined with specialized databases, RefSeq^45^ and ISHAM-ITS reference database (ref. 46).

### Biofilm protocol

Biofilm activity was assessed with an XTT method^47^ and using Resazurin with slight modification on the XTT protocol. Briefly, each strain was grown over night in bottles containing YEPD (Yeast Extract 1%, Peptone 1%, Dextrose 2% - Difco Laboratories, USA) medium, at 30 °C in an orbital shaker at 150–180 rpm and then harvested and centrifuged at 3,000 × *g* for 5 minutes at 4 °C. The supernatant was removed and the pellet was washed twice with PBS. Washed cells were then resuspended in RPMI-1640 medium (Sigma Aldrich), previously warmed at 37 °C, in order to obtain a final density of 1.0 × 10^6 ^cells/mL. 100 μL of this standardized cell suspension were seeded in each selected well of 96-well microtiter plate; the wells on column 12 remained unseeded, in order to act as negative background control for the subsequent steps.

The microtiter plate was closed, sealed and incubated for 24 h at 37 °C. After biofilm formation, the medium in each well was removed carefully with a multi-channel pipette, taking care of not disrupting the biofilm; each well was subsequently washed three times with PBS. After each washing step, the plate was drained in an inverted position by blotting with paper towels.

Using a multichannel pipette, 100 μl of fresh 0.001 mg/mL resazurin solution was added to each well of the drained plated, included the negative control wells, to assess the biofilm formation. After 1 h incubation at 37 °C, the plate was visually inspected to highlight the presence of pink color gradient, resulting from the reduction of the blue dye resazurin to the pink dye resorufin by living biofilm-forming cells. Both systems produced quite similar results. Strains were considered able to form biofilm when a visible color change was detectable.

### Statistical data analysis

#### Contingency analysis and chi squared test

Contingency analysis and chi squared test is the gold standard approach in ecology for the treatment of qualitative non-continuous data, as those treated in this paper. Contingency analysis was carried out according to Legendre & Legendre^26^, as detailed in Supplementary materials and depicted in the four parts of Table S3.

## Additional Information

**How to cite this article**: Corte, L. *et al.* Exploring ecological modelling to investigate factors governing the colonization success in nosocomial environment of *Candida albicans* and other pathogenic yeasts. *Sci. Rep.* **6**, 26860; doi: 10.1038/srep26860 (2016).

## Supplementary Material

Supplementary Information

## Figures and Tables

**Table 1:** Probabilities associated with the χ^2^ statistics calculated on contingency tables.

a) Pisa	Sp. Med.	Surgery	ICU	Gen. Med.	Rehabilit.		d) Pisa	BF	NBF	g) Pisa	BF	NBF
*C. albicans*	0.98	0.84	0.72	0.59	0.47		Sp. Medicine	0.80	0.66	*C. albicans*	0.39	0.13
*C. glabrata*	0.56	0.58	0.90	0.54	0.76		Surgery	0.81	0.68	*C. glabrata*	0.15	0.01
*C. parapsilosis*	0.74	0.93	0.93	0.33	0.69		ICU	1.00	1.00	*C. parapsilosis*	0.39	0.13
*C. tropicalis*	0.15	0.12	0.77	0.62	0.75		Gen. Medicine	0.81	0.68	*C. tropicalis*	0.22	0.03
*Candida spp*	0.20	0.32	0.73	0.43	<0.01		Rehabilitation	0.39	0.13	*Candida spp*	0.08	0.01
*P. kudriavzevii*	0.31	0.60	0.60	0.79	0.92					*P. kudriavzevii*	1.00	0.32
*C. rugosa*	0.39	0.46	0.05	0.71	0.88					*C. rugosa*	1.00	0.16
**b) Udine**	**Gen. Med.**	**ICU**	**Sp. Med.**	**Surgery**	**Oncohemat.**	**Rehabilit.**	**e) Udine**	**BF**	**NBF**	**h) Udine**	**BF**	**NBF**
*C. albicans*	0.77	0.83	0.76	0.22	0.17	0.55	Gen. Medicine	0.85	0.81	*C. albicans*	<0.01	<0.01
*C. glabrata*	0.79	0.52	0.29	0.76	0.64	0.62	ICU	0.82	0.76	*C. glabrata*	<0.01	<0.01
*C. parapsilosis*	0.91	0.97	0.25	0.17	0.67	0.92	Sp. Medicine	0.92	0.89	*C. parapsilosis*	0.04	0.01
*C. tropicalis*	0.77	0.32	0.48	0.20	<0.01	0.59	Surgery	0.31	0.18	*C. tropicalis*	0.72	0.63
*M. guilliermondii*	0.71	0.60	0.46	0.50	0.08	<0.01	Oncohematology	0.26	0.14	*M. guilliermondii*	0.17	0.07
*C. lusitaniae*	0.46	0.76	0.67	0.70	0.79	0.87	Rehabilitation	0.22	0.11	*C. lusitaniae*	0.43	0.30
*C. sake*	0.46	0.76	0.67	0.70	0.79	0.87				*C. sake*	0.43	0.30
*L. jadinii*	0.46	0.76	0.67	0.70	0.79	0.87				*L. jadinii*	0.43	0.30
**c) Udine & Pisa**	**Sp. Med.**	**Surgery**	**ICU**	**Gen. Med.**	**Rehabilit.**	**Oncohemat.**	**f) Udine & Pisa**	**BF**	**NBF**	**j) Udine & Pisa**	**BF**	**NBF**
*C. albicans*	0.96	0.33	0.73	0.90	0.43	0.20	Sp. Medicine	0.65	0.52	*C. albicans*	<0.01	<0.01
*C. glabrata*	0.20	0.66	0.85	0.52	0.69	0.71	Surgery	0.46	0.29	*C. glabrata*	<0.01	<0.01
*C. parapsilosis*	0.32	0.29	0.97	0.84	0.97	0.62	ICU	0.93	0.90	*C. parapsilosis*	0.03	<0.01
*C. tropicalis*	0.33	0.05	0.58	1.00	0.51	<0.01	Gen. Medicine	0.89	0.84	*C. tropicalis*	0.67	0.54
*Candida spp*	0.43	0.08	0.15	0.08	0.07	0.52	Rehabilitation	0.13	0.03	*Candida spp*	0.22	0.08
*P. kudriavzevii*	0.12	0.66	0.70	0.55	0.88	0.83	Oncohematology	0.21	0.07	*P. kudriavzevii*	0.41	0.24
*C. rugosa*	0.49	0.54	<0.01	0.40	0.84	0.76				*C. rugosa*	0.25	0.10
*M. guilliermondii*	0.39	0.45	0.51	0.96	<0.01	0.02				*M. guilliermondii*	0.16	0.04
*C. lusitaniae*	0.62	0.66	0.70	0.27	0.88	0.83				*C. lusitaniae*	0.41	0.24
*C. sake*	0.62	0.66	0.70	0.27	0.88	0.83				*C. sake*	0.41	0.24
*L. jadinii*	0.62	0.66	0.70	0.27	0.88	0.83				*L. jadinii*	0.41	0.24

Legend. Data are probability values associated with the χ^2^ statistics and assess the hypothesis that the relationship between the two descriptors is random. When the probability value is low the null hypothesis of independence is rejected and therefore the combination between the two descriptors is not considered random but caused by some phenomenon. *p* values below 0.10 are reported in boldface; all data have been rounded to the second decimal digit.

Sp. Med: Specialized Medicine; ICU: Intensive Care Unit; Gen. Med.: General Medicine; Rehabilit: Rehabilitation; Oncohaemat.: Oncohaematology; BF: Biofilm Forming; NBF: Non Biofilm Forming.
