# Supplementary material for: Exploring ecological modelling to investigate factors governing the colonization success in nosocomial environment of Candida albicans and other pathogenic yeasts
Source: Sci Rep. 2016 Jun 1;6:26860. doi: 10.1038/srep26860 (PMC4887984; doi:10.1038/srep26860)
Supplement: Supplementary Information [file srep26860-s1.doc]

**TITLE PAGE**

**Exploring ecological modelling to investigate factors governing the colonization success in nosocomial environment of *Candida albicans* and other pathogenic yeasts**

Laura Corte ^1^, Luca Roscini ^1^, Claudia Colabella ^1^, Carlo Tascini ^2^, Alessandro Leonildi ^2^, Emanuela Sozio ^3^, Francesco Menichetti ^2^, Maria Merelli ^4^, Claudio Scarparo ^5^, Wieland Meyer ^6^, Gianluigi Cardinali ^1,7^* and Matteo Bassetti ^4^.

**^1^** Department of Pharmaceutical Sciences-Microbiology, University of Perugia, Borgo 20 Giugno 74, 06121 Perugia, Italy

**^2^** U.O. Malattie Infettive, Azienda Ospedaliera Universitaria Pisana, Via Paradisa 2, Cisanello, 56100 Pisa, Italy

**^3^** U.O. Medicina d’Urgenza (Emergency Medicine Unit) Universitaria, Azienda Ospedaliera Universitaria Pisana, Via Paradisa 2, Cisanello, 56100 Pisa, Italy

**^4^** Infectious Diseases Clinic, Santa Maria Misericordia University Hospital, Piazzale Santa Maria della Misericordia, 15, 33100 Udine, Italy

**^5^** Microbiology Unit, Santa Maria Misericordia University Hospital, Piazzale Santa Maria della Misericordia, 15, 33100 Udine, Italy.

**^6^** Molecular Mycology Research Laboratory, Westmead Hospital, Darcy Road, Westmead, NSW, 2145, Australia

**^7^** CEMIN, Centre of Excellence on Nanostructured Innovative Materials, Department of Chemistry, Biology and Biotechnology, University of Perugia, Via Elce di Sotto 8, 06123 Perugia, Italy.

***Corresponding Author**: Dr. Gianluigi Cardinali

Dept. of Pharmaceutical Sciences – Microbiology

Borgo 20 Giugno, 74

I – 06121 PERUGIA (ITALY)

e.mail: [gianluigi.cardinali@unipg.it](mailto:gianluigi.cardinali@unipg.it)

phone +39 075 585 6478; fax +39 075 585 6470

**Statistical data analysis**

*Contingency analysis and χ^2^ test*

Contingency analysis is the gold standard approach in ecology for the treatment of qualitative non-continuous data, as those treated in this paper. Contingency analysis was carried out in four steps depicted, as an example, in the four parts of Table S3:

1. **Observed data two way contingency table**

Contingency tables reported the first descriptor (e.g. the species) in the first column and the second descriptor (e.g. the various departments) in the first row (**panel a**). Each cell of the table reports the number of occurrences obtained by combining the various states of the two descriptors For instance, in panel a, there are 17 strains isolated in Specialized Medicine departments and belonging to the species *C. albicans*. These data will be hereinafter referred to as “observed data”.

1. **Relative frequency two way table**

Since this table reports absolute observed frequencies, it can be hard to compare with other similar contingency table due to the difference in the total number of observed occurrences. For this reason, absolute frequencies are transformed in relative frequencies with the formula 1 and reported as example in **panel b**.

*Formula 1 RF = (100/n)*AF*

Where RF and AF are the relative and absolute frequencies, respectively, and *n* is the number of observed occurrences, i.e. the sum of all observed values. Relative frequencies will be typically reported as bar or columns graphs in this paper for easy comparisons.

1. **Expected values two way contingency table**

On the basis of the observed data, a table with theoretically expected absolute frequencies was prepared, according to Legendre & Legendre [^25^](#_ENREF_25), using the formula 2

*Formula 2 E = [(row sum) x (column sum)]/n,*

where *E* is the Expected value reported in each cell, *n* is the number of observations, i.e. of the strains in the Observed absolute frequency in panel a and ***row sum*** and ***column sum*** are the sum of strains reported respectively in the row and in the column of crossing in the cell to calculate (**panel c**). These data will be herein referred to as “expected data”, for simplification.

1. **χ^2^ (chi-squared) table**

The statistic test to assess whether there is a significant difference between observed and expected data is the *χ^2^* (chi-squared), which is obtained with formula 3

*Formula 3 χ^2^= (O-E)/E*

Where *O* and *E* indicate the observed and the expected data, respectively. These figures are reported in a fourth panel (**panel d**). The probability (*p-value*) to accept the null hypothesis is typically obtained by comparing the *χ^2^* tables with the actual calculated values, or by performing a *χ^2^* test, resulting directly in the *p-value* to accept or reject the null hypothesis. The *p****-****values* close to 1 indicate that the null hypothesis can be accepted, whereas low (typically 0.1, 0.05 or less) values indicate that indeed the null hypothesis must be rejected and the data combination is significantly different from that theoretically expectable with a random distribution.

**TABLES**

**TABLE S1.** NCAC strains (n = 117) employed in the study.

| **NCAC strains** | | | | | | | | | | | | |
| --- | --- | --- | --- | --- | --- | --- | --- | --- | --- | --- | --- | --- |
| Strain  number | Species | Ward | Origin  of  specimen | BF | City |  | Strain  number | Species | Ward | Origin  of  specimen | BF | City |
| **6381a** | *C. glabrata* | SU | bloodculture | BF | Pi |  | **6595** | *C. parapsilosis* | GM | bloodculture | BF | Ud |
| **6381b** | *C. glabrata* | SU | bloodculture | BF | Pi |  | **6634** | *C. parapsilosis* | SM | bloodculture | BF | Ud |
| **4673** | *C. glabrata* | SM | bloodculture | NBF | Pi |  | **112** | *C. parapsilosis* | GM | bloodculture | BF | Ud |
| **4988** | *C. glabrata* | ICU | bloodculture | NBF | Pi |  | **21** | *C. parapsilosis* | GM | bloodculture | NBF | Ud |
| **5700** | *C. glabrata* | SM | bloodculture | NBF | Pi |  | **25** | *C. parapsilosis* | GM | bloodculture | NBF | Ud |
| **5910** | *C. glabrata* | GM | bloodculture | NBF | Pi |  | **28** | *C. parapsilosis* | GM | bloodculture | NBF | Ud |
| **5976** | *C. glabrata* | ICU | bloodculture | NBF | Pi |  | **68** | *C. parapsilosis* | GM | bloodculture | NBF | Ud |
| **6377** | *C. glabrata* | SU | bloodculture | NBF | Pi |  | **236** | *C. parapsilosis* | GM | bloodculture | NBF | Ud |
| **2685** | *C. glabrata* | GM | bloodculture | BF | Ud |  | **911** | *C. parapsilosis* | GM | bloodculture | NBF | Ud |
| **1032** | *C. glabrata* | SU | bloodculture | NBF | Ud |  | **1489** | *C. parapsilosis* | GM | bloodculture | NBF | Ud |
| **2764** | *C. glabrata* | SU | bloodculture | NBF | Ud |  | **4389** | *C. parapsilosis* | GM | bloodculture | NBF | Ud |
| **4129** | *C. glabrata* | SU | bloodculture | NBF | Ud |  | **5214** | *C. parapsilosis* | GM | bloodculture | NBF | Ud |
| **6018** | *C. glabrata* | SU | bloodculture | NBF | Ud |  | **33** | *C. parapsilosis* | SM | bloodculture | NBF | Ud |
| **54** | *C. glabrata* | GM | bloodculture | NBF | Ud |  | **37** | *C. parapsilosis* | SM | bloodculture | NBF | Ud |
| **293** | *C. glabrata* | GM | bloodculture | NBF | Ud |  | **3605** | *C. parapsilosis* | SM | bloodculture | NBF | Ud |
| **546** | *C. glabrata* | GM | bloodculture | NBF | Ud |  | **5554** | *C. parapsilosis* | SM | bloodculture | NBF | Ud |
| **1425** | *C. glabrata* | GM | bloodculture | NBF | Ud |  | **30** | *C. parapsilosis* | GM | bloodculture | NBF | Ud |
| **1959** | *C. glabrata* | GM | bloodculture | NBF | Ud |  | **38** | *C. parapsilosis* | SM | bloodculture | NBF | Ud |
| **2590** | *C. glabrata* | GM | bloodculture | NBF | Ud |  | **19** | *C. parapsilosis* | O | bloodculture | NBF | Ud |
| **4880** | *C. glabrata* | GM | bloodculture | NBF | Ud |  | **478** | *C. parapsilosis* | O | bloodculture | NBF | Ud |
| **5923** | *C. glabrata* | GM | bloodculture | NBF | Ud |  | **6471** | *C. parapsilosis* | O | bloodculture | NBF | Ud |
| **6070** | *C. glabrata* | GM | bloodculture | NBF | Ud |  | **4111** | *C. parapsilosis* | R | bloodculture | NBF | Ud |
| **6609** | *C. glabrata* | SM | bloodculture | NBF | Ud |  | **52** | *C. parapsilosis* | GM | bloodculture | NBF | Ud |
| **7584** | *C. glabrata* | SM | bloodculture | NBF | Ud |  | **2868** | *C. parapsilosis* | ICU | bloodculture | NBF | Ud |
| **2658** | *C. glabrata* | GM | bloodculture | NBF | Ud |  | **5532** | *C. parapsilosis* | ICU | bloodculture | NBF | Ud |
| **1239** | *C. glabrata* | O | bloodculture | NBF | Ud |  | **5156a** | *C. rugosa* | ICU | bloodculture | NBF | Pi |
| **4128** | *C. glabrata* | R | bloodculture | NBF | Ud |  | **5156b** | *C. rugosa* | ICU | bloodculture | NBF | Pi |
| **3704** | *C. glabrata* | GM | bloodculture | NBF | Ud |  | **3645** | *C. sake* | GM | bloodculture | NBF | Ud |
| **20** | *C. glabrata* | ICU | bloodculture | NBF | Ud |  | **4705** | *C. tropicalis* | SM | bloodculture | BF | Pi |
| **2750** | *C. glabrata* | ICU | bloodculture | NBF | Ud |  | **5500** | *C. tropicalis* | SM | bloodculture | BF | Pi |
| **4960** | *C. glabrata* | ICU | bloodculture | NBF | Ud |  | **5728** | *C. tropicalis* | SM | bloodculture | BF | Pi |
| **5425** | *M. guilliermondii* | GM | bloodculture | NBF | Ud |  | **5945a** | *C. tropicalis* | SM | bloodculture | BF | Pi |
| **910** | *M. guilliermondii* | O | bloodculture | NBF | Ud |  | **5945b** | *C. tropicalis* | SM | bloodculture | BF | Pi |
| **3703** | *M. guilliermondii* | R | bloodculture | NBF | Ud |  | **6184a** | *C. tropicalis* | ICU | peritoneal fluid | BF | Pi |
| **5499** | *P. kudriavzevii* | SM | bloodculture | NBF | Pi |  | **6184b** | *C. tropicalis* | ICU | peritoneal fluid | BF | Pi |
| **6446** | *C. lusitaniae* | GM | bloodculture | NBF | Ud |  | **6548** | *C. tropicalis* | GM | bloodculture | BF | Pi |
| **4616** | *C. parapsilosis* | SM | bloodculture | BF | Pi |  | **8467** | *C. tropicalis* | SM | bloodculture | BF | Pi |
| **4618** | *C. parapsilosis* | SU | bloodculture | BF | Pi |  | **35** | *C. tropicalis* | GM | bloodculture | BF | Ud |
| **4758** | *C. parapsilosis* | ICU | bloodculture | BF | Pi |  | **134** | *C. tropicalis* | O | bloodculture | BF | Ud |
| **4845** | *C. parapsilosis* | SM | bloodculture | BF | Pi |  | **971** | *C. tropicalis* | GM | bloodculture | BF | Ud |
| **5640** | *C. parapsilosis* | ICU | bloodculture | BF | Pi |  | **1557** | *C. tropicalis* | GM | bloodculture | BF | Ud |
| **5944** | *C. parapsilosis* | SM | bloodculture | BF | Pi |  | **2447** | *C. tropicalis* | O | bloodculture | BF | Ud |
| **6510** | *C. parapsilosis* | SU | bloodculture | BF | Pi |  | **3269** | *C. tropicalis* | GM | bloodculture | BF | Ud |
| **6551** | *C. parapsilosis* | ICU | pharyngeal swab | BF | Pi |  | **7036** | *C. tropicalis* | GM | bloodculture | NBF | Ud |
| **5821** | *C. parapsilosis* | ICU | bloodculture | NBF | Pi |  | **7206** | *C. tropicalis* | GM | bloodculture | NBF | Ud |
| **5830** | *C. parapsilosis* | SM | bloodculture | NBF | Pi |  | **4525** | *C. tropicalis* | SM | bloodculture | NBF | Ud |
| **6139** | *C. parapsilosis* | SU | bloodculture | NBF | Pi |  | **22** | *C. tropicalis* | O | bloodculture | NBF | Ud |
| **6509** | *C. parapsilosis* | SM | bloodculture | NBF | Pi |  | **6817** | *C. tropicalis* | O | bloodculture | NBF | Ud |
| **6511** | *C. parapsilosis* | SU | bloodculture | NBF | Pi |  | **12** | *L. jadinii* | GM | bloodculture | NBF | Ud |
| **8333** | *C. parapsilosis* | SM | bone abscess | NBF | Pi |  | **5573** | *Candida spp* | ICU | bloodculture | BF | Pi |
| **29** | *C. parapsilosis* | ICU | bloodculture | BF | Ud |  | **5729** | *Candida spp* | ICU | bloodculture | BF | Pi |
| **1681** | *C. parapsilosis* | SU | bloodculture | BF | Ud |  | **6792/14** | *Candida spp* | SU | bloodculture | BF | Pi |
| **2656** | *C. parapsilosis* | SM | bloodculture | BF | Ud |  | **4663** | *Candida spp* | R | bloodculture | NBF | Pi |
| **4480** | *C. parapsilosis* | GM | bloodculture | BF | Ud |  | **5034** | *Candida spp* | SM | bloodculture | NBF | Pi |
| **4694** | *C. parapsilosis* | SM | bloodculture | BF | Ud |  | **5111** | *Candida spp* | SU | bloodculture | NBF | Pi |
| **4970** | *C. parapsilosis* | GM | bloodculture | BF | Ud |  | **5251** | *Candida spp* | SU | bloodculture | NBF | Pi |
| **6055** | *C. parapsilosis* | GM | bloodculture | BF | Ud |  | **5393** | *Candida spp* | SU | bloodculture | NBF | Pi |
| **6071** | *C. parapsilosis* | SU | bloodculture | BF | Ud |  | **6052** | *Candida spp* | ICU | bloodculture | NBF | Pi |
| **6510** | *C. parapsilosis* | SM | bloodculture | BF | Ud |  |  |  |  |  |  |  |

**Legend.** The abbreviations **BF** and **NBF** stands for “Biofilm Forming” and “Non Biofilm Forming”, respectively. Hospital departments are indicated as follows: **SU** Surgery, **SP** Specialistic Medicine, **GM** General Medicine, **O** Oncohematology, **R** Rehabilitation and **ICU** Intensive Care Unit. Strain numbers are referred to the internal hospital collection from which isolates have been isolated.

**TABLE S2.** *Candida albicans* strains (n = 160) employed in the study.

| ***Candida albicans* strains** | | | | | | | | | | | | |
| --- | --- | --- | --- | --- | --- | --- | --- | --- | --- | --- | --- | --- |
| Strain  number | Species | Ward | Origin  of  specimen | BF | City |  | Strain  number | Species | Ward | Origin  of  specimen | BF | City |
| **4529** | *C. albicans* | SP | bloodculture | BF | Pi |  | **1319** | *C. albicans* | SP | bloodculture | BF | Ud |
| **4541** | *C. albicans* | SP | bloodculture | BF | Pi |  | **1634** | *C. albicans* | SU | bloodculture | BF | Ud |
| **4547** | *C. albicans* | SP | bloodculture | BF | Pi |  | **1682** | *C. albicans* | SU | bloodculture | BF | Ud |
| **4637** | *C. albicans* | SP | bloodculture | BF | Pi |  | **1683** | *C. albicans* | O | bloodculture | BF | Ud |
| **4690** | *C. albicans* | SP | bloodculture | BF | Pi |  | **1684** | *C. albicans* | GM | bloodculture | BF | Ud |
| **4780** | *C. albicans* | SU | bloodculture | BF | Pi |  | **1897** | *C. albicans* | GM | bloodculture | BF | Ud |
| **4846** | *C. albicans* | SP | bloodculture | BF | Pi |  | **2026** | *C. albicans* | SP | bloodculture | BF | Ud |
| **4862** | *C. albicans* | ICU | bloodculture | BF | Pi |  | **2157** | *C. albicans* | ICU | bloodculture | BF | Ud |
| **4897** | *C. albicans* | SP | bloodculture | BF | Pi |  | **2369** | *C. albicans* | GM | bloodculture | BF | Ud |
| **4934** | *C. albicans* | ICU | bloodculture | BF | Pi |  | **2419** | *C. albicans* | GM | bloodculture | BF | Ud |
| **4940** | *C. albicans* | SU | bloodculture | BF | Pi |  | **2431** | *C. albicans* | SP | bloodculture | BF | Ud |
| **4941** | *C. albicans* | SU | bloodculture | BF | Pi |  | **2448** | *C. albicans* | ICU | bloodculture | BF | Ud |
| **5031** | *C. albicans* | ICU | bloodculture | BF | Pi |  | **2655** | *C. albicans* | GM | bloodculture | BF | Ud |
| **5032** | *C. albicans* | SU | bloodculture | BF | Pi |  | **2691** | *C. albicans* | GM | bloodculture | BF | Ud |
| **5033** | *C. albicans* | ICU | bloodculture | BF | Pi |  | **2709** | *C. albicans* | GM | bloodculture | BF | Ud |
| **5079** | *C. albicans* | SP | bloodculture | BF | Pi |  | **2806** | *C. albicans* | SU | bloodculture | BF | Ud |
| **5328** | *C. albicans* | ICU | bloodculture | BF | Pi |  | **2870** | *C. albicans* | GM | bloodculture | BF | Ud |
| **5394** | *C. albicans* | SP | bloodculture | BF | Pi |  | **3089** | *C. albicans* | SP | bloodculture | BF | Ud |
| **5427** | *C. albicans* | SP | bloodculture | BF | Pi |  | **3101** | *C. albicans* | SP | bloodculture | BF | Ud |
| **5701** | *C. albicans* | SP | bloodculture | BF | Pi |  | **3170** | *C. albicans* | SP | bloodculture | BF | Ud |
| **5977** | *C. albicans* | ICU | bloodculture | BF | Pi |  | **3241** | *C. albicans* | GM | bloodculture | BF | Ud |
| **5978** | *C. albicans* | SU | bloodculture | BF | Pi |  | **3288** | *C. albicans* | GM | bloodculture | BF | Ud |
| **6183** | *C. albicans* | GM | bloodculture | BF | Pi |  | **3338** | *C. albicans* | GM | bloodculture | BF | Ud |
| **6371** | *C. albicans* | ICU | bloodculture | BF | Pi |  | **3393** | *C. albicans* | ICU | bloodculture | BF | Ud |
| **6376** | *C. albicans* | SU | bloodculture | BF | Pi |  | **3584** | *C. albicans* | GM | bloodculture | BF | Ud |
| **6378** | *C. albicans* | SP | bloodculture | BF | Pi |  | **3638** | *C. albicans* | GM | bloodculture | BF | Ud |
| **6379a** | *C. albicans* | ICU | bloodculture | BF | Pi |  | **3716** | *C. albicans* | SU | bloodculture | BF | Ud |
| **6379b** | *C. albicans* | ICU | bloodculture | BF | Pi |  | **3765** | *C. albicans* | GM | bloodculture | BF | Ud |
| **6380** | *C. albicans* | SU | bloodculture | BF | Pi |  | **3833** | *C. albicans* | ICU | bloodculture | BF | Ud |
| **6493** | *C. albicans* | SU | bloodculture | BF | Pi |  | **3839** | *C. albicans* | SP | bloodculture | BF | Ud |
| **6505** | *C. albicans* | GM | bloodculture | BF | Pi |  | **3963** | *C. albicans* | GM | bloodculture | BF | Ud |
| **6506a** | *C. albicans* | SP | bloodculture | BF | Pi |  | **3983** | *C. albicans* | GM | bloodculture | BF | Ud |
| **6506b** | *C. albicans* | SP | bloodculture | BF | Pi |  | **4117** | *C. albicans* | O | bloodculture | BF | Ud |
| **6507a** | *C. albicans* | SU | bloodculture | BF | Pi |  | **4238** | *C. albicans* | GM | bloodculture | BF | Ud |
| **6507b** | *C. albicans* | SU | bloodculture | BF | Pi |  | **4311** | *C. albicans* | GM | bloodculture | BF | Ud |
| **6508** | *C. albicans* | GM | bloodculture | BF | Pi |  | **4441** | *C. albicans* | GM | bloodculture | BF | Ud |
| **6549** | *C. albicans* | ICU | bloodculture | BF | Pi |  | **4512** | *C. albicans* | GM | bloodculture | BF | Ud |
| **6550** | *C. albicans* | GM | bloodculture | BF | Pi |  | **4537** | *C. albicans* | GM | bloodculture | BF | Ud |
| **6552** | *C. albicans* | SP | bloodculture | BF | Pi |  | **4575** | *C. albicans* | GM | bloodculture | BF | Ud |
| **6792** | *C. albicans* | ICU | bloodculture | BF | Pi |  | **4777** | *C. albicans* | ICU | bloodculture | BF | Ud |
| **8158** | *C. albicans* | SU | vascular prosthesis | BF | Pi |  | **4823** | *C. albicans* | GM | bloodculture | BF | Ud |
| **6381** | *C. albicans* | SU | bloodculture | BF | Pi |  | **4881** | *C. albicans* | GM | bloodculture | BF | Ud |
| **8468** | *C. albicans* | SP | bloodculture | BF | Pi |  | **4882** | *C. albicans* | SU | bloodculture | BF | Ud |
| **7731A3** | *C. albicans* | SP | bloodculture | BF | Pi |  | **4940** | *C. albicans* | SP | bloodculture | BF | Ud |
| **8158/C** | *C. albicans* | SU | vascular prosthesis | NBF | Pi |  | **5045** | *C. albicans* | R | bloodculture | BF | Ud |
| **1** | *C. albicans* | SU | bloodculture | BF | Ud |  | **5151** | *C. albicans* | GM | bloodculture | BF | Ud |
| **2** | *C. albicans* | GM | bloodculture | BF | Ud |  | **5193** | *C. albicans* | GM | bloodculture | BF | Ud |
| **3** | *C. albicans* | GM | bloodculture | BF | Ud |  | **5213** | *C. albicans* | GM | bloodculture | BF | Ud |
| **4** | *C. albicans* | GM | bloodculture | BF | Ud |  | **5316** | *C. albicans* | GM | bloodculture | BF | Ud |
| **6** | *C. albicans* | GM | bloodculture | BF | Ud |  | **5434** | *C. albicans* | SP | bloodculture | BF | Ud |
| **11** | *C. albicans* | ICU | bloodculture | BF | Ud |  | **5435** | *C. albicans* | GM | bloodculture | BF | Ud |
| **13** | *C. albicans* | GM | bloodculture | BF | Ud |  | **5454** | *C. albicans* | SU | bloodculture | BF | Ud |
| **15** | *C. albicans* | SP | bloodculture | BF | Ud |  | **5512** | *C. albicans* | SP | bloodculture | BF | Ud |
| **16** | *C. albicans* | SP | bloodculture | BF | Ud |  | **5656** | *C. albicans* | SU | bloodculture | BF | Ud |
| **18** | *C. albicans* | GM | bloodculture | BF | Ud |  | **5908** | *C. albicans* | GM | bloodculture | BF | Ud |
| **23** | *C. albicans* | SP | bloodculture | BF | Ud |  | **6060** | *C. albicans* | SP | bloodculture | BF | Ud |
| **24** | *C. albicans* | SP | bloodculture | BF | Ud |  | **6069** | *C. albicans* | GM | bloodculture | BF | Ud |
| **27** | *C. albicans* | GM | bloodculture | BF | Ud |  | **6075** | *C. albicans* | GM | bloodculture | BF | Ud |
| **31** | *C. albicans* | SU | bloodculture | BF | Ud |  | **6104** | *C. albicans* | GM | bloodculture | BF | Ud |
| **32** | *C. albicans* | GM | bloodculture | BF | Ud |  | **6127** | *C. albicans* | SU | bloodculture | BF | Ud |
| **34** | *C. albicans* | O | bloodculture | BF | Ud |  | **6732** | *C. albicans* | GM | bloodculture | BF | Ud |
| **36** | *C. albicans* | ICU | bloodculture | BF | Ud |  | **7130** | *C. albicans* | GM | bloodculture | BF | Ud |
| **39** | *C. albicans* | ICU | bloodculture | BF | Ud |  | **7142** | *C. albicans* | SU | bloodculture | BF | Ud |
| **40** | *C. albicans* | ICU | bloodculture | BF | Ud |  | **7146** | *C. albicans* | SU | bloodculture | BF | Ud |
| **51** | *C. albicans* | SU | bloodculture | BF | Ud |  | **7205** | *C. albicans* | GM | bloodculture | BF | Ud |
| **53** | *C. albicans* | SU | bloodculture | BF | Ud |  | **7286** | *C. albicans* | GM | bloodculture | BF | Ud |
| **146** | *C. albicans* | GM | bloodculture | BF | Ud |  | **6916** | *C. albicans* | SU | bloodculture | NBF | Ud |
| **499** | *C. albicans* | GM | bloodculture | BF | Ud |  | **26a** | *C. albicans* | SU | bloodculture | NBF | Ud |
| **506** | *C. albicans* | SP | bloodculture | BF | Ud |  | **2034** | *C. albicans* | SP | bloodculture | NBF | Ud |
| **604** | *C. albicans* | SU | bloodculture | BF | Ud |  | **1960** | *C. albicans* | GM | bloodculture | NBF | Ud |
| **617** | *C. albicans* | SP | bloodculture | BF | Ud |  | **4536** | *C. albicans* | GM | bloodculture | NBF | Ud |
| **699** | *C. albicans* | SU | bloodculture | BF | Ud |  | **3112** | *C. albicans* | SP | bloodculture | NBF | Ud |
| **703** | *C. albicans* | SU | bloodculture | BF | Ud |  | **4022** | *C. albicans* | SP | bloodculture | NBF | Ud |
| **846** | *C. albicans* | GM | bloodculture | BF | Ud |  | **4326** | *C. albicans* | GM | bloodculture | NBF | Ud |
| **906** | *C. albicans* | SU | bloodculture | BF | Ud |  | **7400** | *C. albicans* | R | bloodculture | NBF | Ud |
| **991** | *C. albicans* | SU | bloodculture | BF | Ud |  | **603** | *C. albicans* | GM | bloodculture | NBF | Ud |
| **1026** | *C. albicans* | SP | bloodculture | BF | Ud |  | **14** | *C. albicans* | ICU | bloodculture | NBF | Ud |
| **1224** | *C. albicans* | SU | bloodculture | BF | Ud |  | **4686** | *C. albicans* | ICU | bloodculture | NBF | Ud |
| **1305** | *C. albicans* | SU | bloodculture | BF | Ud |  | **6508** | *C. albicans* | SP | bloodculture | NBF | Ud |
| **1318** | *C. albicans* | GM | bloodculture | BF | Ud |  | **26b** | *C. albicans* | O | bloodculture | NBF | Ud |

**Legend.** The abbreviations **BF** and **NBF** stands for “Biofilm Forming” and “Non Biofilm Forming”, respectively. Hospital departments are indicated as follows: **SU** Surgery, **SP** Specialistic Medicine, **GM** General Medicine, **O** Oncohematology, **R** Rehabilitation and **ICU** Intensive Care Unit. Strain numbers are referred to the internal hospital collection from which they are been isolated.

**TABLE S3.** Example of contingency analysis carried out with data from the Pisa Hospital.

| **a** |  | |  |  |  |  |
| --- | --- | --- | --- | --- | --- | --- |
|  | | **Sp. Medicine** | **Surgery** | **ICU** | **Gen. Medicine** | **Rehabilitation** |
| ***C. albicans*** | | 17 | 13 | 11 | 4 | 0 |
| ***C. glabrata*** | | 2 | 3 | 2 | 1 | 0 |
| ***C. parapsilosis*** | | 6 | 4 | 4 | 0 | 0 |
| ***C. tropicalis*** | | 6 | 0 | 2 | 1 | 0 |
| ***Candida spp*** | | 1 | 4 | 3 | 0 | 1 |
| ***P. kudriavzevii*** | | 1 | 0 | 0 | 0 | 0 |
| ***C. rugosa*** | | 0 | 0 | 2 | 0 | 0 |
|  | |  |  |  |  |  |
|  | |  |  |  |  |  |
| **b** | |  |  |  |  |  |
|  | | **Sp. Medicine** | **Surgery** | **ICU** | **Gen. Medicine** | **Rehabilitation** |
| ***C. albicans*** | | 19.32% | 14.77% | 12.50% | 4.55% | 0.00% |
| ***C. glabrata*** | | 2.27% | 3.41% | 2.27% | 1.14% | 0.00% |
| ***C. parapsilosis*** | | 6.82% | 4.55% | 4.55% | 0.00% | 0.00% |
| ***C. tropicalis*** | | 6.82% | 0.00% | 2.27% | 1.14% | 0.00% |
| ***Candida spp*** | | 1.14% | 4.55% | 3.41% | 0.00% | 1.14% |
| ***P. kudriavzevii*** | | 1.14% | 0.00% | 0.00% | 0.00% | 0.00% |
| ***C. rugosa*** | | 0.00% | 0.00% | 2.27% | 0.00% | 0.00% |
| **c** | |  |  |  |  |  |
|  | | **Sp. Medicine** | **Surgery** | **ICU** | **Gen. Medicine** | **Rehabilitation** |
| ***C. albicans*** | | 16.875 | 12.272 | 12.272 | 3.068 | 0.511 |
| ***C. glabrata*** | | 3.000 | 2.181 | 2.181 | 0.545 | 0.090 |
| ***C. parapsilosis*** | | 5.250 | 3.818 | 3.818 | 0.954 | 0.159 |
| ***C. tropicalis*** | | 3.375 | 2.454 | 2.454 | 0.613 | 0.102 |
| ***Candida spp*** | | 3.375 | 2.454 | 2.454 | 0.613 | 0.102 |
| ***P. kudriavzevii*** | | 0.375 | 0.272 | 0.272 | 0.068 | 0.011 |
| ***C. rugosa*** | | 0.750 | 0.545 | 0.545 | 0.136 | 0.022 |
|  | |  |  |  |  |  |
| **d** | |  |  |  |  |  |
|  | | **Sp. Medicine** | **Surgery** | **ICU** | **Gen. Medicine** | **Rehabilitation** |
| ***C. albicans*** | | 0.001 | 0.043 | 0.131 | 0.282 | 0.511 |
| ***C. glabrata*** | | 0.333 | 0.302 | 0.015 | 0.378 | 0.090 |
| ***C. parapsilosis*** | | 0.107 | 0.008 | 0.008 | 0.954 | 0.159 |
| ***C. tropicalis*** | | 2.040 | 2.454 | 0.084 | 0.243 | 0.102 |
| ***Candida spp*** | | 1.671 | 0.973 | 0.121 | 0.613 | **7.880** |
| ***P. kudriavzevii*** | | 1.041 | 0.272 | 0.272 | 0.068 | 0.011 |
| ***C. rugosa*** | | 0.750 | 0.545 | 3.878 | 0.136 | 0.022 |
|  |  | |  |  |  |  |

**Legend.** **Panel a.** absolute frequencies contingency table; **panel b**. relative frequencies contingency table; **panel c**. expected absolute values contingency table, **panel d.** *χ^2^* table . *χ^2^* significance: > 3.84 for *p=0.05*, > 6.64 for *p=0.01*.

The *χ^2^* significance, often referred to as table *χ^2^* significance, indicated the statistical significance for the table as a whole.
